# Estrogen antagonizes ASIC1a-induced chondrocyte mitochondrial stress in rheumatoid arthritis

**DOI:** 10.1186/s12967-022-03781-1

**Published:** 2022-12-03

**Authors:** Zhuoyan Zai, Yayun Xu, Xuewen Qian, Zihan Li, Ziyao Ou, Tao Zhang, Longfei Wang, Yian Ling, Xiaoqing Peng, Yihao Zhang, Feihu Chen

**Affiliations:** 1 School of Pharmacy, Anhui Medical University, No. 81 Mei Shan Road, Shu Shan District, Hefei, 230032 Anhui China; 2 School of Public Health, Anhui Medical University, No. 81 Mei Shan Road, Shu Shan District, Hefei, 230032 Anhui China; 3 Inflammation and Immune Mediated Diseases Laboratory of Anhui Province, Anhui Institute of Innovative Drugs, No. 81 Mei Shan Road, Shu Shan District, Hefei, 230032 Anhui China; 4 Anhui Province Key Laboratory of Major Autoimmune Diseases, Anhui Medical University, No. 81 Mei Shan Road, Shu Shan District, Hefei, 230032 Anhui China; 5 Department of Obstetrics and Gynecology, The First Affiliated Hospital of Anhui Medical University, 218 Jixi Road, Hefei, 230022 Anhui China; 6 Department of Toxicology, School of Public Health, Anhui Medical University, Hefei, China; 7 Key Laboratory of Environmental Toxicology of Anhui Higher Education Institutes, Hefei, China

**Keywords:** Rheumatoid arthritis, ASIC1a, Calcium influx, Mitochondrial stress, Estrogen

## Abstract

**Background:**

Destruction of articular cartilage and bone is the main cause of joint dysfunction in rheumatoid arthritis (RA). Acid-sensing ion channel 1a (ASIC1a) is a key molecule that mediates the destruction of RA articular cartilage. Estrogen has been proven to have a protective effect against articular cartilage damage, however, the underlying mechanisms remain unclear.

**Methods:**

We treated rat articular chondrocytes with an acidic environment, analyzed the expression levels of mitochondrial stress protein HSP10, ClpP, LONP1 by q-PCR and immunofluorescence staining. Transmission electron microscopy was used to analyze the mitochondrial morphological changes. Laser confocal microscopy was used to analyze the Ca^2+^, mitochondrial membrane potential (Δψm) and reactive oxygen species (ROS) level. Moreover, ASIC1a specific inhibitor Psalmotoxin 1 (Pctx-1) and Ethylene Glycol Tetraacetic Acid (EGTA) were used to observe whether acid stimulation damage mitochondrial function through Ca^2+^ influx mediated by ASIC1a and whether pretreatment with estrogen could counteract these phenomena. Furthermore, the ovariectomized (OVX) adjuvant arthritis (AA) rat model was treated with estrogen to explore the effect of estrogen on disease progression.

**Results:**

Our results indicated that HSP10, ClpP, LONP1 protein and mRNA expression and mitochondrial ROS level were elevated in acid-stimulated chondrocytes. Moreover, acid stimulation decreased mitochondrial membrane potential and damaged mitochondrial structure of chondrocytes. Furthermore, ASIC1a specific inhibitor PcTx-1 and EGTA inhibited acid-induced mitochondrial abnormalities. In addition, estrogen could protect acid-stimulated induced mitochondrial stress by regulating the activity of ASIC1a in rat chondrocytes and protects cartilage damage in OVX AA rat.

**Conclusions:**

Extracellular acidification induces mitochondrial stress by activating ASIC1a, leading to the damage of rat articular chondrocytes. Estrogen antagonizes acidosis-induced joint damage by inhibiting ASIC1a activity. Our study provides new insights into the protective effect and mechanism of action of estrogen in RA.

## Introduction

Rheumatoid arthritis (RA) is an autoimmune disease characterized by joint lesions. The global incidence of RA ranges from 0.5 to 1% with sex differences [1, 2]. Women have a higher incidence than men, with the incidence significantly higher in postmenopausal than in premenopausal women [3]. A variety of extracellular harmful factors, such as inflammation, pH changes, and hypoxia, lead to the damage and death of articular chondrocytes, which is the direct cause of articular cartilage destruction and joint deformity. Clinically, RA treatment can effectively relieve inflammatory symptoms but has little effect on articular cartilage damage [4]. Estrogen replacement therapy protects the joint cartilage and reduces symptoms such as joint pain in clinical settings, suggesting that it has a protective effect on RA articular cartilage damage [5, 6]. In vivo, ovariectomized (OVX) animals tended to have higher rates of RA and more severe disease states than OVX sham animals [3]. However, the protective mechanism of estrogen in articular cartilage remains unknown.

Acidification of the extracellular microenvironment is a shared feature of inflammatory diseases and tumors, which mediates the malignant phenotype of tumor cells and leads to inflammation [7–9]. Studies have shown that inflammation leads to increased anaerobic glycolysis and promotes the secretion and accumulation of lactic acid, resulting in a significant decrease in the pH of the RA joint cavity [10, 11]. RA joint imaging shows that the degree of synovial acidification is positively correlated with joint destruction [12, 13]. Acid-sensing ion channel 1a (ASIC1a) is an important acid sensor with permeability to Ca^2+^ and Na^+^, which is involved in various diseases such as RA, cancer, and stroke in response to extracellular acidification. We previously found that ASIC1a is highly expressed in articular chondrocytes of adjuvant arthritis (AA) model rats compared to that in normal rats [14, 15]. Moreover, activation of ASIC1a induces rat chondrocyte death in response to a low-pH extracellular microenvironment and promotes the destruction of articular cartilage in an AA model [16]. Therefore, ASIC1a is a key molecule that mediates the destruction of RA articular cartilage; however, its mechanism needs to be further clarified.

Ca^2+^ is an important intracellular secondary messenger that mediates a series of physiological and pathological changes. The repeated opening of Ca^2+^ channels located in the plasma membrane or endoplasmic reticulum mediates the entry of a large amount of Ca^2+^ into the cytoplasm, thereby causing Ca^2+^ overload. It is widely recognized that intracellular Ca^2+^ overload leads to cell death. As an H^+^-activated Ca^2+^ channel, ASIC1a induces rat chondrocyte death by mediating extracellular Ca^2+^ influx in an acidic microenvironment, which contributes to articular cartilage damage. In vivo, the ASIC1a-specific inhibitor Psalmotoxin 1 (Pctx-1) had a significant protective effect on rat articular cartilage [17, 18]. Our previous research found that estrogen promotes the degradation of the ASIC1a protein via the autophagy-lysosomal pathway and further prevents acidosis-induced chondrocyte death [19, 20]. However, the mechanism by which ASIC1a-induced Ca^2+^ influx leads to chondrocyte death remains to be explored.

Mitochondrial stress comprises a series of responses triggered by harmful stimuli that lead to mitochondrial dysfunction. Mitochondria are key energy-providing organelles that determine cell fate. Under conditions of mitochondrial stress, the cell state changes from energetic to dying. Ca^2+^ overload is one of the primary causes of mitochondrial stress. High concentrations of Ca^2+^ from the cytoplasm are taken up by mitochondria, which disrupts mitochondrial Ca^2+^ homeostasis and causes a series of stress reactions. When the mitochondrial stress response breaks the threshold, it leads to the release of apoptotic factors, such as cytochrome c, apoptosis-inducing factor, and caspase-9, from the mitochondrial intermembrane space to the cytoplasm, which triggers apoptosis [21]. Mitochondrial stress is a key pathogenic factor that mediates cell death and tissue damage and exacerbates various disease conditions. Moreover, mitochondrial stress has an important pathogenic role [22, 23]. Therefore, it is particularly important to elucidate the role and mechanism of mitochondrial stress in cartilage destruction in RA.

In this study, we found that extracellular acidification induced mitochondrial stress via ASIC1a-mediated Ca^2+^ influx in chondrocytes. Activation of ASIC1a by extracellular acidification induced mitochondrial reactive oxygen species (ROS) production, impaired mitochondrial membrane potential and membrane structure. Moreover, estrogen antagonized extracellular acidification-induced mitochondrial stress by inhibiting the activity of ASIC1a. Furthermore, estrogen had a dose-dependent effect on resisting disease progression and attenuating joint damage in OVX AA rats. In conclusion, our study demonstrated that ASIC1a mediates mitochondrial stress in chondrocytes and the protective effect of estrogen on RA articular cartilage.

## Materials and methods

### Animals

Female Sprague–Dawley (SD) rats, aged 7–8 weeks weighing 180–220 g, were provided by the Experimental Animal Center of the Anhui Medical University (Anhui, Hefei, China). The rats were housed in a room at 37 ℃ and 75% humidity and allowed free access to standard pelleted food and water. All experiments conformed to the ethical principles and guidelines approved by the Animal Experimental Ethics Review Committee of the Anhui Medical University.

### Isolation and culture of articular chondrocytes

Joint tissues were removed from the rats. Under sterile conditions, knee cartilage tissues were minced into small pieces (approximately 1 mm^3^) in a cell culture dish containing phosphate buffered saline (PBS) with 100 IU/mL penicillin, and 100 μg/mL streptomycin. Next, 0.2% type II collagenase was added to digest the tissues at 37 ℃ for 5 h. The chondrocytes were carefully separated after digestion using a 70 μm filter and centrifuged at 200 × g for 10 min. Then, the chondrocytes were attached to the wall of the cell culture flask and cultured in DMEM/high glucose medium supplemented with 10% fetal bovine serum (FBS), 100 IU/mL penicillin, and 100 μg/mL streptomycin. The cultures were cultured at 37 ℃ at 5% CO_2_ and sub-cultured 2–5 times.

### Adjuvant arthritis induction and treatment

Rats were ovariectomized or removed adipose tissue around the ovaries before molding. The OVX and sham-operated rats received intradermal immunization into the left hind metatarsal footpad with 0.1 mL heat-killed mycobacteria (10 mg/mL) suspended in Complete Freund’s adjuvant (CFA, Chondrex Inc., USA). On the 20th day after immunization, the rats were randomly allocated for in vivo experiments (n = 6): the normal group, the model group, the OVX and estradiol (E2)-injected group (E2 was injected at 0.1, 0.2, and 0.3 mg/kg) (Sigma, USA, once every 3 days, total eight times), OVX and triamcinolone acetonide (TA)-treated group (positive control, 1 mg/kg, Selleck, USA, intra-articular injection, once every 3 days, total eight times), and the sham group.

### Immunohistochemistry and hematoxylin and eosin staining and toluidine blue staining

Rat secondary ankle joints were harvested, fixed in 4% paraformaldehyde, soaked in 10% ethylenediaminetetraacetic acid, and embedded in paraffin. Immunohistochemistry (IHC) staining was performed using the SP9000 IHC reagent kit (ZSGB Bio, China) according to the manufacturer’s protocol. Hematoxylin and eosin (H&E) staining and toluidine blue staining were performed using the H&E staining kit (Beyotime, China) and toluidine blue according to the manufacturer’s protocol. Serial sections were examined using a digital pathology slide scanner (3DHISTECH, Hungary).

### Ca^2+^ imaging

Cytosolic Ca^2+^ levels in isolated rat articular chondrocytes were measured using the fluorescent Ca^2+^ indicator Fluo-3 AM (Dojindo Laboratories). Briefly, the chondrocytes were washed three times with D-Hanks’ solution, incubated with 2.5 μM Fluo3-AM for 30 min at 37 ℃ on coverslips, washed three times, and incubated in Hank's for another 30 min. Fluo-3 AM was quantitated by confocal laser scanning fluorescence microscopy (Zeiss, Germany), at an excitation of 488 nm and an emission of 525 nm.

### Mitochondrial membrane potential measurement

Mitochondrial membrane potential (Δψm) was measured using a mitochondrial membrane potential assay kit with JC-1 (BestBio, China), according to the manufacturer’s protocol. Cells were grown in glass-bottomed dishes. After treatment with the different doses for the different times, the cells were cultured with 5 μg/mL JC-1 for 25 min at 37 °C in a humidified 5% CO_2_ incubator. Fluorescence intensity was determined using a confocal microscope (Zeiss, Germany). J-monomer was measured with excitation at 514 nm and emission at 529 nm, and J-aggregates with excitation at 585 nm and emission at 590 nm.

### Quantitative real-time polymerase chain reaction

Total RNA was extracted using the TRIzol Reagent (AG Scientific, China) and reverse transcribed into complementary DNA (cDNA) using Evo M-MLV RT Premix for qPCR (AG Scientific, China) according to the manufacturer’s instructions. The SYBR Green Premix Pro Taq HS qPCR kit (AG Scientific, China) was used for analysis. The primer sequences used are shown in Table 1.

**Table 1:** Primer sequences for real-time PCR

Gene	Forward primer sequence (5′ → 3′)	Reverse primer sequence (3′ → 5′)
β-actin	CCCATCTATGAGGGTTACGC	TTTAATGTCACGCACGATTTC
LONP1	GGAGACAAGTTGCGCATGAT	CCTCTTTCTTGCCTCGCTTC
Hsp10	GAGTATTGGTTGAAAGGAGTG	TGACAGGCTGAATCTCTCC
ClpP	AGGCACCTAAGGACTTGACC	TTCTAGCCCAGCAGAGGAAG

### Western blotting

Total protein was isolated from chondrocytes at various time points using RIPA (Beyotime, China). The lysates were mixed with loading buffer, subjected to SDS-PAGE (80 V, 30 min, then 120 V, 60 min), and electro-transferred to a polyvinylidene fluoride (PVDF) membrane (Millipore Corp., Billerica, MA, USA). The PVDF membranes were incubated in Tris-buffered saline with Tween 20 (TBS-T) containing 5% skim milk for 2 h to block the membrane. Next, the membranes were incubated with antibodies against ASIC1a (Abcam, 1:1000) and β-actin (Abcam, 1:1000) overnight at 4 °C. The next day, the membranes were incubated with horseradish peroxidase (HRP)-conjugated goat anti-mouse or anti-rabbit secondary antibodies for 1 h after washing. Finally, the membranes were imaged using an enhanced chemiluminescence kit (ECL-plus, Thermo Fisher Scientific). Densitometric analysis was performed using the ImageJ software.

### Immunofluorescence staining

For determination of localization of mitochondrial stress proteins in chondrocytes, a modified Mito-Tracker Red CMXRos kit (Beyotime, China) was used according to manufacturer’s instructions to label mitochondria, at an excitation of 579 nm and an emission of 599 nm. Cultured cells were washed thrice with PBS and fixed in 4% paraformaldehyde for 20 min. Cells were first treated with CMXRos for 20 min. The cells were permeabilized for 10 min with 0.1% Triton X-100 and blocked with 5% BSA for 30 min at room temperature. Next, the cells were stained with anti-HSP10, anti-ClpP, or anti-LONP1 antibodies (1:500; Bioss, Beijing, China) with 1% BSA overnight at 4 °C. The next day, the cells were washed three times with PBS for 5 min, incubated with the corresponding fluorescent-labeled secondary antibody (FITC, 1:200 Boster, Wuhan) for 1 h in the dark, washed three times with PBS for 5 min, and counterstained with 4′,6-diamidino-2-phenylindole (DAPI) for 5 min. Images were captured using a confocal microscope (Zeiss, Germany). FITC was analyzed with excitation at 495 nm and emission at 591 nm. DAPI was analyzed with excitation at 353 nm and emission at 465 nm.

### Reactive oxygen species (ROS) analysis

Cellular mitochondrial ROS generation was detected using a mitochondrial ROS-specific staining kit (Bestbio, China). Chondrocytes were treated with 1 mL fluorescent probe and incubated at 37 °C for 30 min. After washing with PBS in glass-bottom dishes, the cells were analyzed using a confocal microscope (Zeiss, Germany), at an excitation of 518 nm and an emission of 580 nm.

### Transmission electron microscopy (TEM)

Rat articular chondrocytes were harvested, fixed in 2.5% glutaraldehyde, and stored at 4 °C for 12 h. Then the chondrocytes were washed in 0.1 M phosphate buffer (pH 7.2) for three times. After further fixated in 1% osmium tetroxide for 2 h, dehydrated by a graded series of ethanol (30%, 50%, 70%, 80%, 90%, 95% and 100%) for about 15 to 20 min at each step, transferred to the mixture of alcohol and iso-amyl acetate (v:v = 1:1) for 1 h, and then embedded in epoxy resin, ultrathin slices (70 nm) were cut from each sample by using a ultramicrotome (leica UC-7, Germany). The sections were then dehydrated, embedded, and counterstained for TEM observation (JEM1400, Japan).

### Statistical analysis

GraphPad Prism 7 (GraphPad Software, USA) was used to analyze the data, which are presented as mean ± standard deviation (SD). A one-way analysis of variance (ANOVA), followed by the least significant difference (LSD) post-hoc test, was used to compare the means of three or more groups to determine whether they differ significantly from one another. *P* < 0.05 was considered statistically significant, with *P < 0.05, **P < 0.01, and ***P < 0.001 indicating the levels of significance.

## Results

### Acidic microenvironment induced mitochondrial stress by activating ASIC1a in articular chondrocytes

**Fig. 1:**
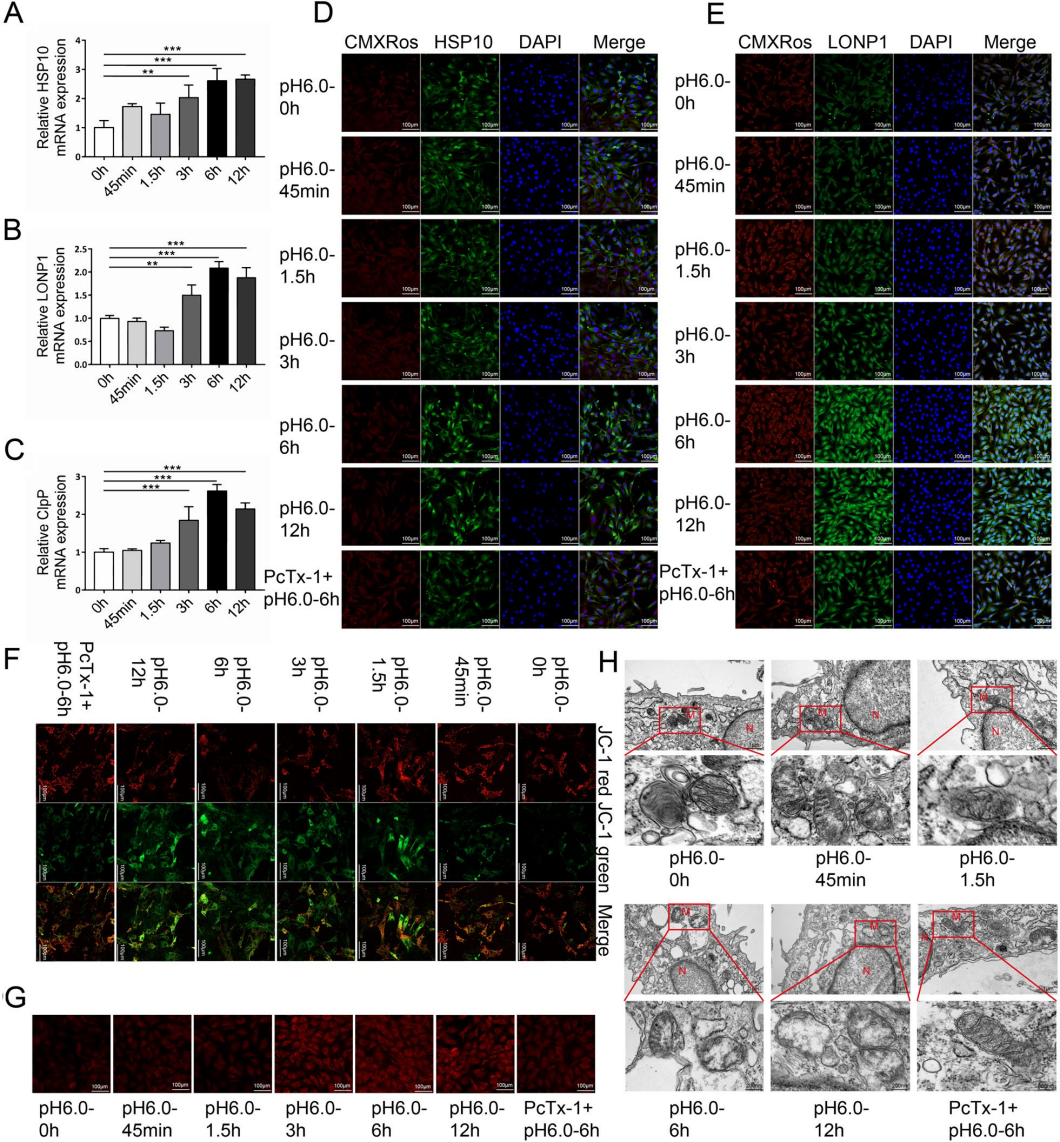
ASIC1a induced mitochondrial stress in primary rat articular chondrocytes. **A**–**C** *Hsp10*, *Lonp1*, and *ClpP* mRNA levels in acid-treated chondrocytes quantified by real-time q-PCR. **D**, **E** Immunofluorescence analysis of HSP10 and LONP1 expression in chondrocytes after pretreatment with pH 6.0 and pH 6.0 + PcTx-1. The scale bars are 20 μm. **F** Representative confocal microscopy images of chondrocytes stained with JC-1 after pretreatment with pH 6.0 and pH 6.0 + PcTx-1. Red fluorescence indicates normal ΔΨm with JC-1 aggregate form in the mitochondria, while green fluorescence reflects the JC-1 monomer, indicative of ΔΨm loss. The scale bars are 20 μm. **G** The release of mitochondrial ROS in chondrocytes was measured after pre-treatment with pH 6.0 and pH 6.0 + PcTx-1. The scale bars are 20 μm. **H** Effects of acid on the mitochondria in chondrocytes, as assessed by TEM. Ultrastructural analysis of the mitochondria. Key: M, mitochondria; N, nucleus; Scale bar, 1 μm (top panels) and 200 nm (bottom panels). Data are presented as the mean ± SD of three independent experiments. *P < 0.05, **P < 0.01, ***P < 0.001

We explored the involvement of acidosis in mitochondrial stress by treating rat articular chondrocytes with an acidic environment (pH 6.0) for 45 min, 1.5, 3, 6, and 12 h. Acid stimulation caused a time-dependent increase in the expression of *Hsp10*, *ClpP*, and *Lonp1* mRNA levels in rat articular chondrocytes (Fig. 1A–C). Immunofluorescence staining showed that the expression of HSP10, ClpP, and LONP1 proteins significantly increased under the above conditions (Figs. 1D, E and 4F). We evaluated the effects of acid stimulation time on the regulation of mitochondrial membrane potential (Δψm) in chondrocytes using the JC-1 assay. The ratio of red to green fluorescence significantly decreased with increasing stimulation time in the group with pH 6.0, reflecting a decrease in Δψm in chondrocytes and the occurrence of apoptosis (Fig. 1F). Acid stimulation of rat articular chondrocytes led to an increase in mitochondrial ROS production, as indicated by mitochondrial ROS fluorescence intensity increasing substantially compared to that in vehicle-treated cells in a time-dependent manner (Fig. 1G). We performed an ultrastructural analysis of the cells using TEM. In the blank control group, mitochondrial structures had a regular shape, the membrane structure was clear, and the cristae were intact. Representative images of acid stimulation groups showed detailed mitochondrial impairments, including swollen mitochondria, cristae loss, and collapsed membranes, and the degree of damage was time-dependent (Fig. 1H).

When rat articular chondrocytes were pretreated with the ASIC1a specific inhibitor PcTx-1 in the acid solution, the acid-induced increase in *Hsp10*, *ClpP*, and *Lonp1* mRNA was diminished (Fig. 4A–C), and immunofluorescence staining showed a significant decrease in the expression of HSP10, ClpP, and LONP1 (Figs. 1D, E and 4F). Moreover, the reduced membrane potential, ROS level and damaged mitochondrial structure were significantly restored (Fig. 1F–H).

### ASIC1a mediated mitochondrial stress by triggering Ca^2+^ influx

**Fig. 2:**
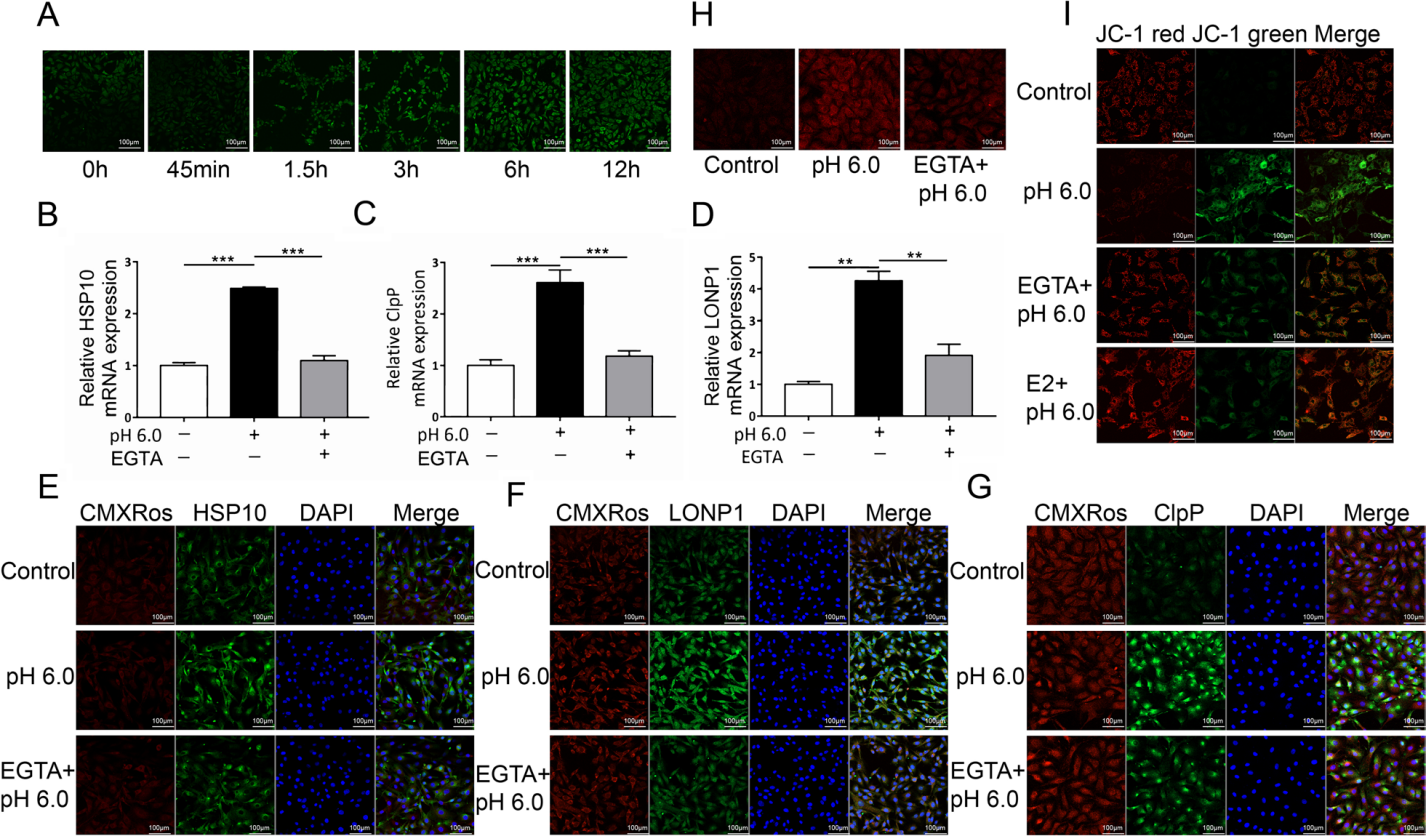
ASIC1a upregulated mitochondrial stress by mediating Ca^2+^ influx. **A** The effect of ASIC1a on Ca^2+^ in chondrocytes at a pH of 6.0, as detected by calcium imaging. The scale bars are 20 μm. **B**–**D** *Hsp10*, *Lonp1*, and *ClpP* mRNA levels in acid-mediated chondrocytes after EGTA pretreatment quantified by real-time q-PCR. **E**–**G** HSP10, LONP1, and ClpP expression in acid-mediated chondrocytes after EGTA pretreatment quantified immunofluorescence. The scale bars are 20 μm. **H** Measurements of ROS released by mitochondria in chondrocytes after pre-treatment with EGTA. **I** Representative confocal microscopy images of chondrocytes stained with JC-1 after pre-treatment with E2 and EGTA. Red fluorescence indicates normal ΔΨm with JC-1 aggregates in the mitochondria, and green fluorescence reflects the JC-1 monomer, indicative of ΔΨm loss. The scale bars are 20 μm. Data are presented as the mean ± SD of three independent experiments. *P < 0.05, **P < 0.01, ***P < 0.001

**Fig. 3:**
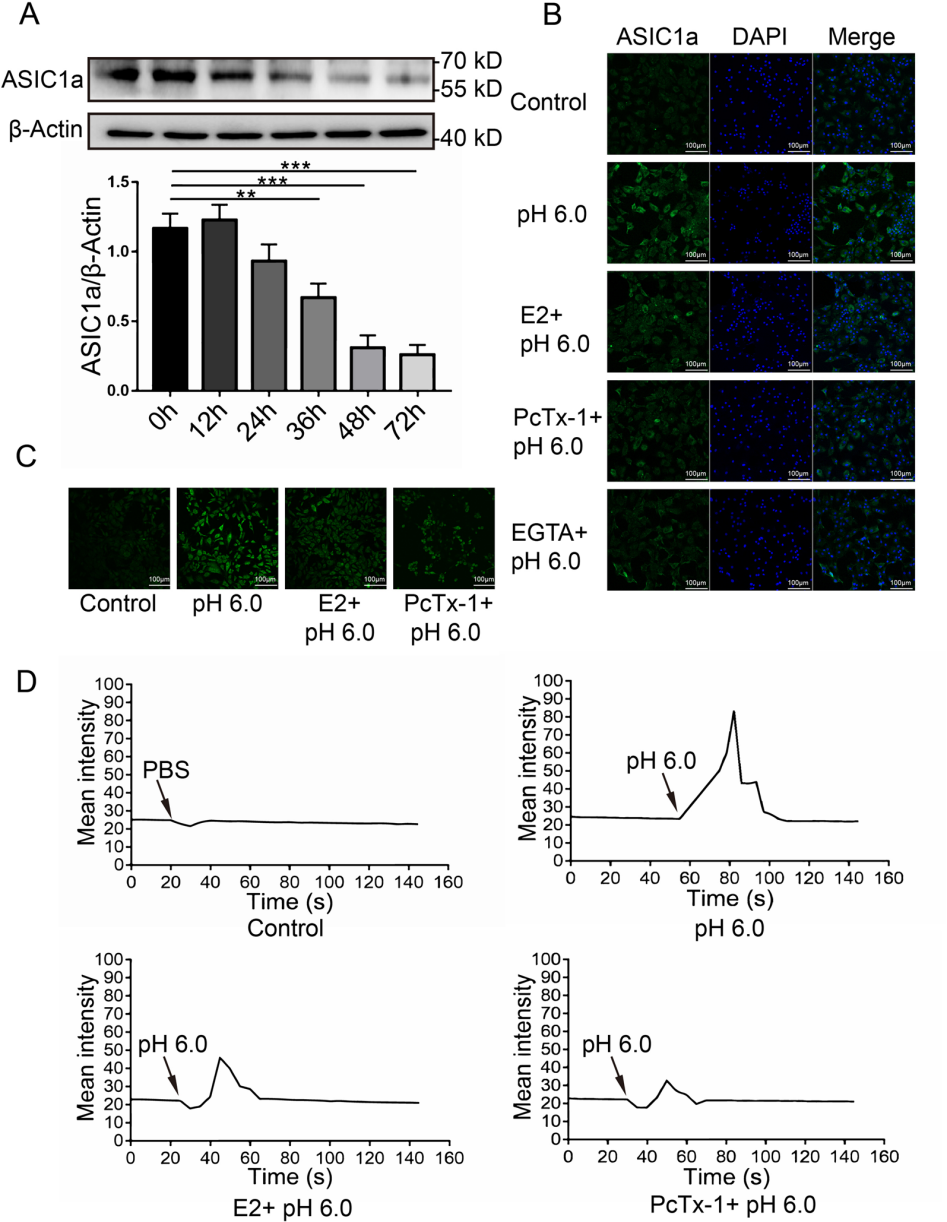
Estrogen decreased ASIC1a activity in articular chondrocytes. **A** The expression of ASIC1a decreased in a time-dependent manner following stimulation with 1000 nmol/mL estradiol for 0–48 h in chondrocytes. **B** Immunofluorescence analysis of ASIC1a expression in chondrocytes treated with PcTx-1, E2, and EGTA. The scale bars are 20 μm. **C** Calcium imaging detected the effect of ASIC1a on Ca^2+^ in chondrocytes treated with E2, PcTx-1. The scale bars are 20 μm. **D** Calcium imaging revealed ASIC1a-mediated Ca^2+^ influx with E2, PcTx-1. Data are presented as the mean ± SD of three independent experiments. *P < 0.05, **P < 0.01, ***P < 0.001

The effect of ASIC1a activation on intracellular Ca^2+^ concentration in an acidic solution (pH 6.0) was investigated using fluorescent Ca^2+^ imaging. The Ca^2+^ concentration gradually increased in chondrocytes treated with an acid solution for 45 min, 1.5, 3, 6, 12, and 24 h. After 6 h, the fluorescence intensity essentially stabilized, whereas in the presence of PcTx-1 or estradiol (E2), the acid-induced increase in Ca^2+^ concentration was diminished (Figs. 2A and 3C). Moreover, compared with the pH 6.0 group, EGTA pretreatment inhibited the acid-induced expression of HSP10, ClpP, and LONP1 at both the protein and mRNA level, which increased in response to acid stimulation in chondrocytes (Fig. 2B–G). We then examined the level of ROS activity in the mitochondria. Under acidic conditions, ROS levels was increased. Following EGTA pretreatment, a significant decrease in ROS levels in the mitochondria was observed (Fig. 2H). Additionally, pretreatment of chondrocytes with EGTA or E2 resulted in a significant increase in ΔΨm compared to that in the pH 6.0 group (Fig. 2I).

### Estrogen inhibited ASIC1a activity in articular chondrocytes

To determine whether E2 regulates ASIC1a channel activity, chondrocytes were treated with E2 for 0, 12, 24, 36, 48, and 72 h, respectively. The results showed that E2 reduced the protein level of ASIC1a in a time-dependent manner and ASIC1a level was stable after 48 h (Fig. 3A). Immunofluorescence staining of ASIC1a in chondrocytes demonstrated that the fluorescence intensity of ASIC1a was decreased with E2 treatment for 48 h compared to the control. Moreover, pretreatment with PcTx-1 and EGTA also reduced the fluorescence intensity of ASIC1 (Fig. 3B). Laser confocal microscopy showed that pretreatment with E2 or PcTx-1 suppressed acid-induced Ca^2+^ elevation (Fig. 3C, D).

### Estrogen antagonized ASIC1a-induced mitochondrial stress

**Fig. 4:**
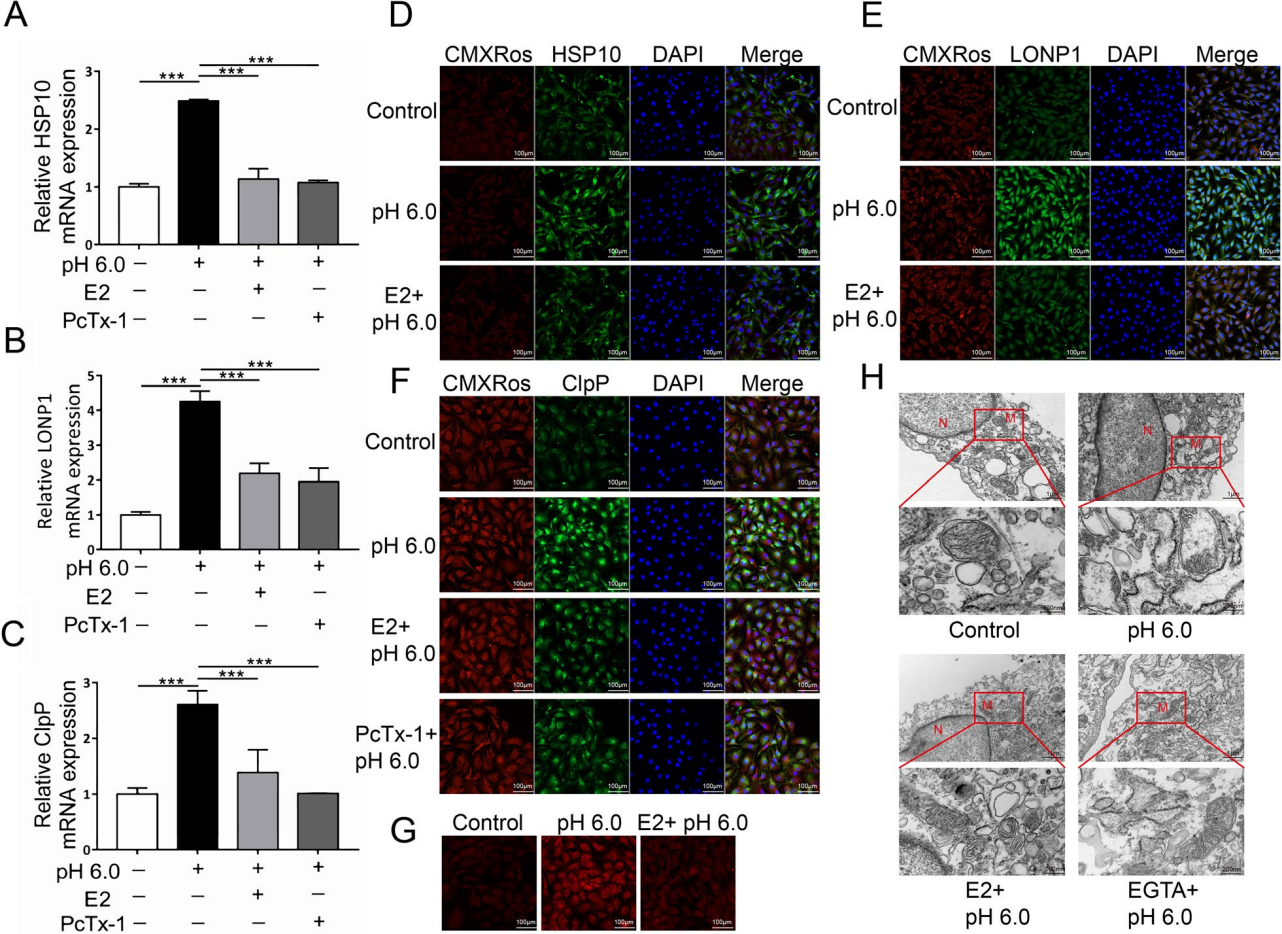
Estrogen antagonized ASIC1a-induced mitochondrial stress. **A**–**C** Quantitative PCR detection of *Hsp10*, *Lonp1*, and *ClpP* mRNA levels in chondrocytes pretreated with E2 and PcTx-1, followed by stimulation with pH 6.0. **D**–**F** Immunofluorescence analysis of HSP10, LONP1and ClpP expression in chondrocytes treated with E2. The scale bars are 20 μm. **G** Measurements of ROS released by mitochondria in chondrocytes after pre-treatment with E2. The scale bars are 20 μm. **H** Effects of E2 on mitochondrial structural damage in ASIC1a-induced chondrocytes as assessed by transmission electron microscopy. Ultrastructural analysis of the mitochondria. Key: M, mitochondria; N, nucleus; Scale bar,1 μm (top panels) and 200 nm (bottom panels). Data are presented as the mean ± SD of three independent experiments. *P < 0.05, **P < 0.01, ***P < 0.001

To investigate whether estrogen has a protective effect on ASIC1a-induced mitochondrial stress, chondrocytes were treated with E2 and PcTx-1. The results showed that, compared with the pH 6.0 group, E2 and PcTx-1 pretreatment inhibited the ASIC1a-induced expression of HSP10, ClpP, and LONP1 at both the mRNA and protein levels (Fig. 4A–F and Fig. 1D, E), which increased in response to acid stimulation in chondrocytes. We then examined the level of ROS activity in the mitochondria. It was observed that the pH 6.0 treatment increased the ROS level, while E2 pretreatment had the opposite effect (Fig. 4G). Moreover, regarding mitochondrial morphological changes, TEM demonstrated that E2 pretreatment decreased acidosis-induced articular chondrocyte mitochondrial injury (Fig. 4H).

### Estrogen protected articular cartilage by antagonizing mitochondrial stress in AA rats

**Fig. 5:**
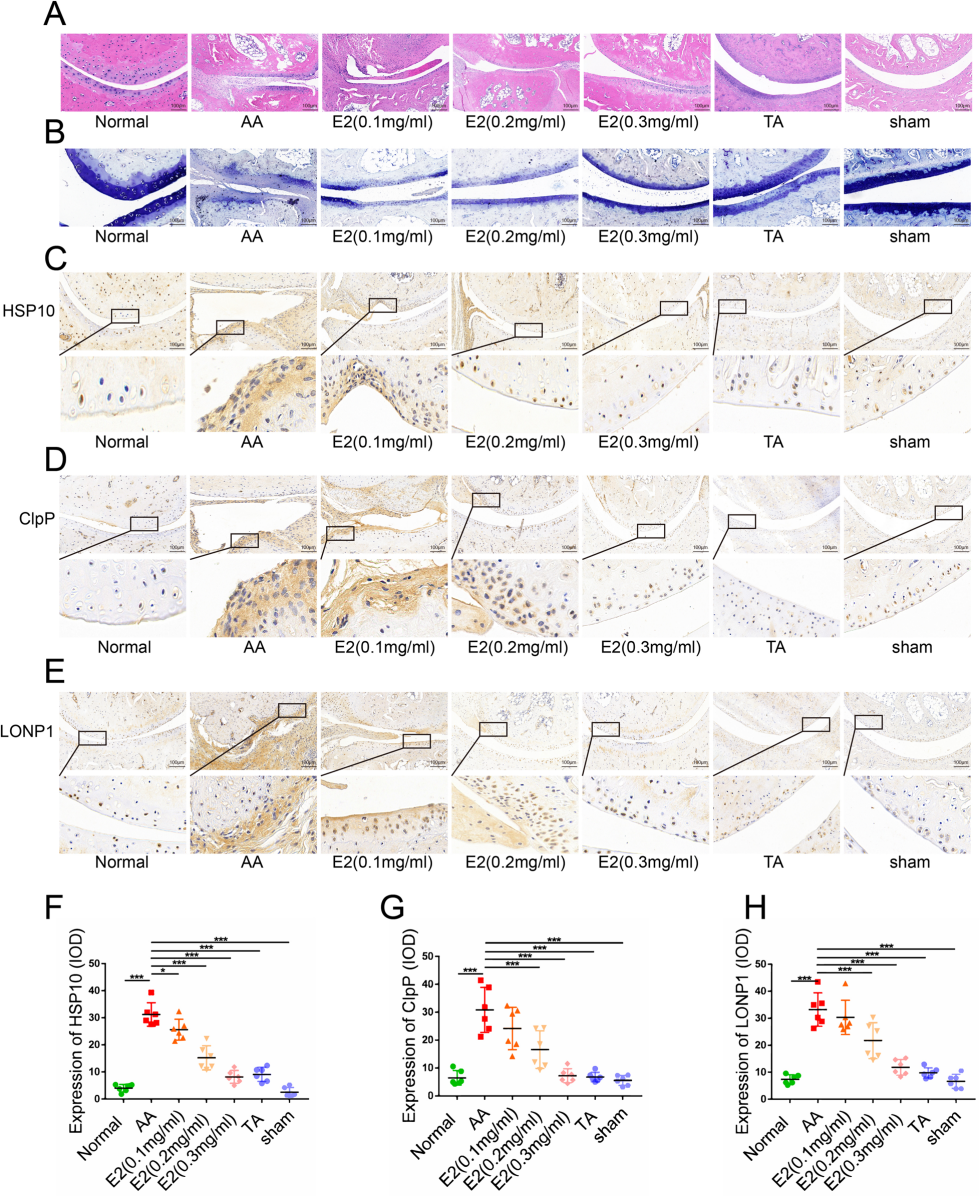
Estradiol treatment improved the histopathological features in ovariectomized AA rats in vivo. Representative micrographs of **A** H&E-stained histological sections (n = 6 per group) and **B** toluidine blue-stained histological sections (n = 6 per group) of the joints of ovariectomized AA rats. Representative micrographs and quantitative analysis of the immunohistochemical detection of **C**, **F** HSP10 expression (n = 6 per group), **D**, **G** ClpP expression (n = 6 per group), and **E**, **H** LONP1 expression (n = 6 per group). *P < 0.05, **P < 0.01, ***P < 0.001

To eliminate the interference of circulating estrogen in the body, the rats were ovariectomized. H&E staining showed synovial hyperplasia as well as cartilage and bone erosion in the cartilage of AA rats (Fig. 5A). Toluidine blue staining showed bone erosion in AA rats compared to the normal group (Fig. 5B). These results indicated that the AA rat models were successfully established. H&E and toluidine blue staining showed that E2 significantly inhibited the development of arthritis in AA rats and reduced cartilage damage and inflammatory infiltration in a dose-dependent manner. To assess the effect of E2 modulation on mitochondrial stress in articular cartilage, we conducted immunohistochemical staining for ClpP, LONP1, and HSP10. The reduction of ClpP, LONP1, and HSP10 by 0.3 mg/kg of E2 was more pronounced than that of 0.1 mg/kg E2 and the solvent groups (Fig. 5C–H).

## Discussion

**Fig. 6:**
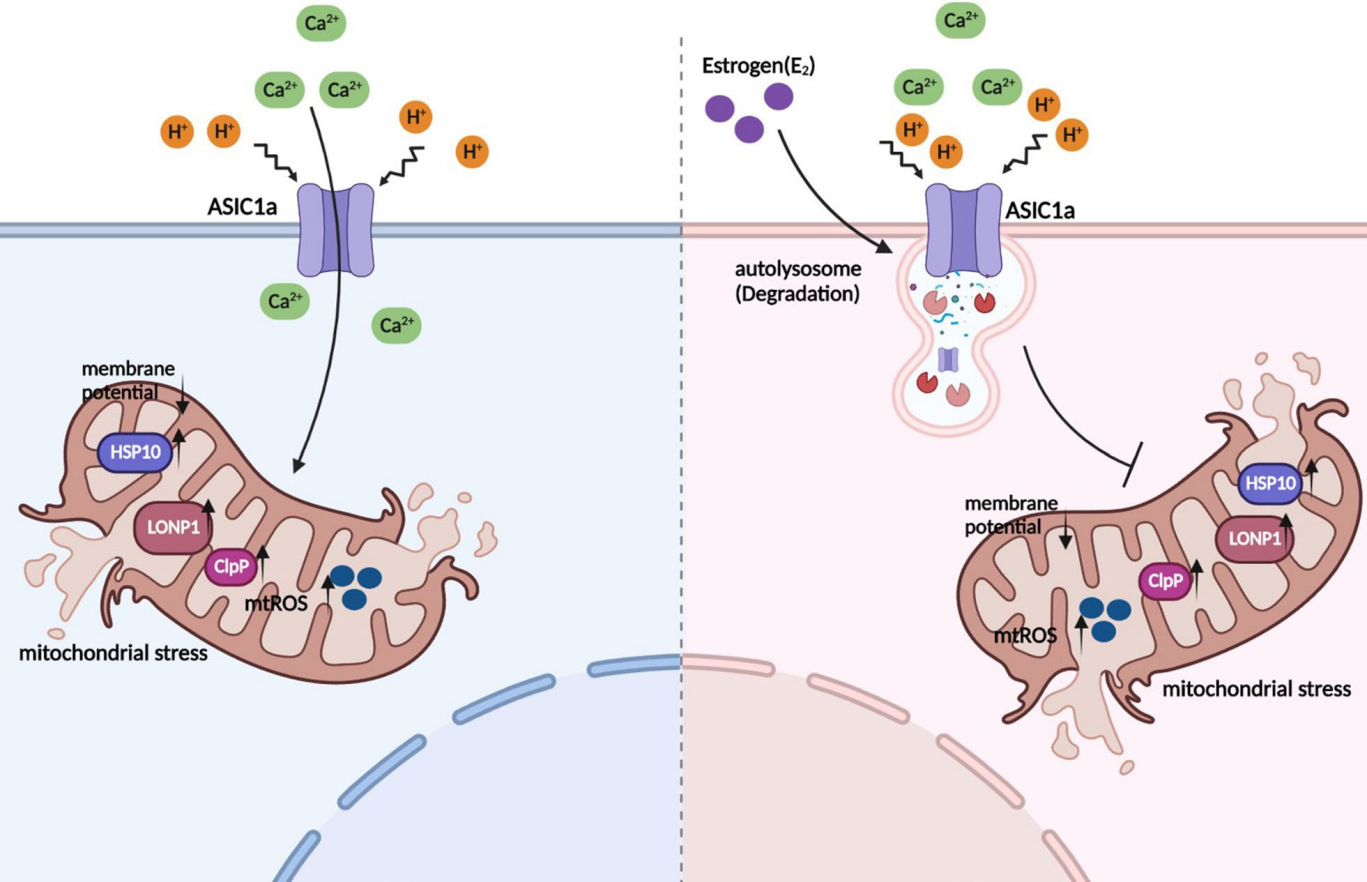
Schematic diagram of the molecular mechanism of estrogen antagonizes ASIC1a-induced mitochondrial stress

Most current research on the pathogenesis of RA focuses on the hyperproliferation of the synovium, while articular cartilage damage has received little attention. Damage to the articular cartilage is an important feature of RA and an urgent clinical problem to be solved [24]. A moderate to strong positive correlation was observed between the degree of cartilage damage and chondrocyte apoptosis [25–27]. A high rate of apoptosis in cartilage would result in matrix degradation, preventing chondrocyte apoptosis, which would be a valid target for modulating articular cartilage destruction. Although numerous studies have sought to elucidate the mechanism of chondrocyte apoptosis, the specific determinants have not yet been fully clarified. Mitochondria are pivotal players in the intrinsic pathway of apoptosis, increased ROS levels, decreased membrane potential, and influence calcium uptake [28, 29]. In this study, we found that ASIC1a was activated by acidic conditions, which is consistent with previously published findings. The major finding of this study was that extracellular acidification activates ASIC1a to mediate calcium influx, which leads to (I) damaged mitochondrial structures, (II) enhanced generation of mitochondrial ROS, (III) decreased mitochondrial membrane potential, and (IV) increased mitochondrial stress protein release. In addition, we also demonstrated that estrogen (I) inhibited ASIC1a activity, (II) resulting in a lack of activation of mitochondrial stress, and consequently (III) attenuates cartilage damage (Fig. 6). These findings imply that ASIC1a could be a potential therapeutic target for preventing cartilage damage and controlling RA progression.

The doses of E2 (0.1, 0.2, and 0.3 mg/kg) and TA (1 mg/kg) used in the present study were selected according to previous studies. Specifically, it has been reported that subcutaneous injection of 0.1 mg/kg E2 reduced the degree of cartilaginous degeneration in an OVX rat model of postmenopausal osteoarthritis [30]. More recently, our previous study has demonstrated that intramuscular injection of E2 (0.1, 0.2 and 0.3 mg/kg) reduced cartilage damage in OVX AA rats [19]. In terms of TA, intra-articular injections with TA (40 mg) are a common treatment used in patients with RA in clinic [31–33]. The dose of rats (1 mg/kg) in the present study was calculated according to the conversion of animal dose to human equivalent doses based on body surface area. Moreover, our previous study has shown that intramuscular injection of TA (1 mg/kg, once every 3 days, total eight times) alleviated synovial hyperplasia, bone erosion, and inflammatory cell infiltration in AA rats [17].

Acidosis is implicated in various diseases, including cancer, atherosclerosis, neurodegenerative disorders, and chronic autoimmune inflammatory diseases [34]. Studies have suggested that lactate plays an important role in protein synthesis, intermediate metabolism, cell growth, and reproduction [35–37]. The normal pH of the extracellular fluid is usually maintained in a range of 7.3–7.4. Acidosis is caused by the accumulation of lactic acid at the inflammatory site [38, 39]. Extracellular acidification is a key factor for cell death [40, 41]. Similarly, acidification of the extracellular environment is a characteristic feature of RA [10, 11, 42]. ASIC1a, a Na^+^ and Ca^2+^-permeable channel, is activated by acid and is involved in the pathogenesis of RA. ASIC1a induces pathophysiological effects in various diseases. It mediates cardiac ischemia–reperfusion injury (IRI), and polymorphisms in its genetic locus are associated with ischemic disease, the therapeutic blockade of ASIC1a reduces cell death, fibrosis, and adverse remodeling, and improves cardiac function [43]. Studies indicate that ASIC1a, which senses the extracellular pH drop triggered by neuronal physiological L-lactate uptake, transmits a Ca^2+^ response that is propagated to mitochondria to enhance lactate catabolism and mitochondrial respiration [44, 45]. We previously demonstrated that ASIC1a expression was higher in AA rat articular cartilage than in that of normal rats. Moreover, extracellular acidosis could activate ASIC1a in rat articular cartilage, ultimately contributing to articular chondrocyte injury via Ca^2+^ overload [15, 16, 46].

Mitochondria play an important role in pro-inflammatory signaling. Moreover, inflammation initiates apoptosis. In our previous study, we found that acidosis induced apoptosis of endplate chondrocytes. This process translocates cytochrome C from the mitochondria to the cytoplasm and activates intracellular caspase-3 and caspase-9, which are classical initiation sites of apoptosis [47]. Mitochondrial stress has long been identified as a possible precursor of apoptosis, and ROS generated by mitochondria during abnormal oxidative metabolism often acts as a trigger for apoptosis. Excessive accumulation of ROS leads to damage to mitochondrial DNA (mtDNA) and mitochondrial structure, and leads to mitochondrial malfunction, which can be repaired by mitochondrial autophagy. However, excessive damage usually affects the whole cell’s physiological activity through the caspase-dependent pathway described above [48–50]. Recent studies have suggested that the Bcl-2-related ovarian killer protein (BOK) may be involved in regulating the mitochondrial pathway of apoptosis through a pathway independent of Bcl-2-like protein 4/Apoptosis regulator Bcl-2 (BAX/BCl-2) [51]. Interestingly, in contrast to the classical process of oxidative stress, the process by which acidification stimulation modulates mitochondrial stress through the accumulation of Ca^2+^ has not been reported. Ca^2+^ is one of the most important signaling secondary messengers in cells, and changes in Ca^2+^ concentration mediate nearly all rapid events that lead to changes in cell function. The mitochondrial surface uses a mitochondrial calcium uniporter (MCU) and a mitochondrial Na^+^/Ca^2+^ exchanger (mNCX) to control Ca^2+^ homeostasis in the mitochondria. Of these, mitochondrial Ca^2+^ uptake 1 (MICU1) senses cytosolic Ca^2+^ concentrations and controls its intake [52–54]. Ca^2+^ homeostasis is important for mitochondrial function, and moderate increases in Ca^2+^ concentration promote the activation of several oxidases of the citric acid cycle (e.g., isocitrate dehydrogenase and oxoglutarate dehydrogenase) and ATP release [55, 56]. Mitochondrial redox metabolism is inevitably accompanied by the production of ROS, and whether the increased level of oxidative metabolism caused by excessive Ca^2+^ accumulation is sufficient for mitochondrial ROS accumulation requires further study. The inhibition of acid-induced mitochondrial stress using inhibitors of ASIC1a and calcium chelators seems to provide some evidence for this hypothesis. However, whether acidification stimulation affects the calcium homeostasis capacity of mitochondria as well as on the cells themselves, which together contribute to this result, requires follow-up research.

As ASIC1a mediates extracellular Ca^2+^ influx, we investigated whether extracellular acidification affects mitochondrial function in rat articular chondrocytes. Specifically, we were interested in the effects and consequences of Ca^2+^ uptake by mitochondria. Owing to the vital physiological roles of mitochondria, which are essential for apoptosis, strategies capable of inhibiting mitochondrial damage pathways in a drug-controllable manner are particularly promising for RA treatment. Previous studies have demonstrated that estradiol induced ASIC1a protein degradation through the estrogen-related receptor-alpha (Esrra) and G-protein coupled estradiol receptor 1 (GPER1) receptors, the mechanisms of which might be related to the Esrra-AMPK-ULK1 and PI3K-GPER1-AKT-mTOR signaling pathways, to protect chondrocytes and rats with AA from acid-induced apoptosis, damage, and autophagy[19, 20]. Estradiol treatment led to the downregulation of ASIC1a proteins in a time- and dose-dependent manner. We further explored whether estrogen regulates the ASIC1a ion channel function and found that treatment with E2 suppressed acid-induced Ca^2+^ elevation, indicating that E2 reduced ASIC1a channel activity. Furthermore, E2 attenuated mitochondrial stress by decreasing ASIC1a channel activity.

It has been observed that ischemia, inflammation, and hypoxia cause extracellular acidosis [42, 57]. The pH value of the synovial fluid in RA patients may fall to 6.0 because RA is a chronic inflammatory condition [11, 12]. Moreover, it has been shown that low synovial fluid pH can disrupt cartilage homeostasis and be linked to radiographic joint degeneration in RA patients [13, 58]. Considering chondrocytes, the only type of cell found in articular cartilage, are essential in the pathogenesis of arthritis and are greatly influenced by the local pH [59], acid-stimulated chondrocyte has been widely used as a cell model to study the pathogenesis of RA [16, 60, 61]. However, since RA is a systemic autoimmune disease, articular cartilage in vivo is affected by a variety of factors, including acidification microenvironment. Therefore, in order to better simulate the extracellular microenvironment of articular cartilage in RA patients, it may be a reasonable strategy to directly use the joint fluid of RA patients in acute phase to stimulate chondrocytes, and then explore the role of ASIC1a in the pathogenesis of RA. Additionally, in the study, type II collagenase digestion was used to separate primary rat articular chondrocytes, which is a commonly used method in the isolation of chondrocytes [62]. Despite being a moderate digestive enzyme, type II collagenase still causes cell damage.

Rats with AA are commonly employed as experimental animal models of polyarthritis to study the pathogenesis of RA and imitate human RA [63]. It has been discovered that rats with AA had lower pH levels in the synovial fluid than animals without AA [16]. This animal model was chosen for the current study because AA generates significant systemic inflammation that leads to severe joint edema and remodeling. However, this model mainly involves inflammation in the articulation and focuses on chondrocyte pathophysiology, while excluding the autoimmune spectrum of RA. Since RA is an autoimmune condition, future studies examining the function of ASIC1a in the pathogenesis of RA should make use of autoimmunity-based RA models, such as collagen-induced arthritis (CIA) or K/BxN animal models. In the present study, repeated intra-articular injection of estradiol or triamcinolone may increase the risk of cartilage damage [64]. More study and investigation should be done on the usage of long-acting medications to decrease the number of injections. More importantly, long-term use of estrogen has been associated with increased risk of breast cancer, thrombosis, and possibly also stroke for the majority of postmenopausal RA patients [65–68]. Whether the local injection of estrogen used in this study can achieve higher efficacy and reduce more adverse reactions needs further study.

In order to clarify the role of estrogen and Ca^2+^ in the development and treatment of RA, their dynamic level fluctuations should be identified at various time intervals. However, this was not implemented in this study, which should be regarded as a limitation. It is worth noting that several studies have investigated the levels of estrogen in arthritis and/or OVX rats. Specifically, our recent study has indicated that the serum estrogen levels were significantly elevated in OVX rats with AA compared to sham rats (rats receiving surgery to remove adipose tissue around ovaries) with AA [19]. Moreover, compared to the OVX rats with AA receiving solvent treatment, the serum estrogen levels were also significantly increased in OVX rats with AA receiving estrogen treatment [19]. Similarly, another study has demonstrated that ovariectomy could reduce the plasma levels of estrogen and intraperitoneal injection of estrogen increased the serum estrogen levels in a rat model of OA [30]. These findings suggested that the serum estrogen level may play a crucial role in the occurrence and development of arthritis and more attention should be paid to the serum level of estrogen in patients with arthritis, especially menopausal women.

## Conclusions

In this study, we showed that extracellular acidification induces mitochondrial stress via ASIC1a-mediated Ca^2+^ influx in chondrocytes, whereas estrogen antagonizes mitochondrial stress by inhibiting ASIC1a activity. These findings imply that ASIC1a could be a potential therapeutic target for preventing cartilage damage and controlling RA progression. New administration mode of estrogen may be considered as a potential therapeutic strategy for RA treatment.

## Data Availability

The data sets used during the current study are available from the corresponding author on reasonable request.
